# “I’d probably trip over it because it’s bumpy”: A qualitative exploration of the lived experiences of ambulatory children with cerebral palsy walking in challenging environments

**DOI:** 10.1371/journal.pone.0337316

**Published:** 2025-12-03

**Authors:** Rebecca L. Walker, Thomas D. O’Brien, Gabor J. Barton, Bernie Carter, David M. Wright, Richard J. Foster

**Affiliations:** 1 Research Institute for Sport and Exercise Sciences, Liverpool John Moores University, Liverpool, United Kingdom; 2 Faculty of Health, Social Care and Medicine, Edge Hill University, Ormskirk, United Kingdom; 3 North West Movement Analysis Centre, Alder Hey Children’s NHS Foundation Trust, Liverpool, United Kingdom; IRCCS Medea: Istituto di Ricovero e Cura a Carattere Scientifico Eugenio Medea, ITALY

## Abstract

**Background:**

Children with cerebral palsy experience regular falls, but their lived experiences of falls in the real-world are unknown. Understanding the perspectives of children and parents is important to gain deeper insight into how falls happen in real-world environments, especially since typical walking analyses are carried out over level-ground and may overlook everyday challenges to balance (e.g., uneven pavements when walking to school). Walk-along interviews can generate rich insights into children’s everyday life by discussing experiences while walking.

**Aim:**

The Walk-Along Project aimed to explore lived experiences of ambulatory children with cerebral palsy to determine challenging walking environments that increase fall risk day-to-day, using walk-along interviews.

**Methods:**

Twelve ambulatory children with cerebral palsy (12 ± 3 years old, 6 hemiplegia, 6 diplegia) and their parents took part in an outdoor walk-along interview. Previous fall experiences and everyday challenging environments that may increase fall risk were discussed. Action cameras and microphones captured walking environments and conversations, which were later synchronised, transcribed and analysed in NVivo using interpretive description.

**Results:**

Two themes were generated (‘places where trips and falls occur’ and ‘things children do to control falls and manage consequences’) plus five subthemes (‘walking on bumpy and unstable ground’, ‘taking care, walking slower and avoiding places’, ‘distracting environments are dangerous environments’, ‘close calls and falls’, and ‘managing consequences and concerns’). The most common environment suggested to increase fall risk was uneven surfaces (e.g., grass potholes) with distractions (e.g., dogs barking).

**Conclusions:**

The Walk-Along Project reveals novel insights about places that increase fall risk in ambulatory children with cerebral palsy, beyond what is currently known. The importance of considering both environmental challenges (e.g., uneven surfaces) and sensory challenges (e.g., distractions) is highlighted through children’s lived experiences. Future work should consider how interacting factors (e.g., distractions in uneven environments) increase fall-risk in ambulatory children with cerebral palsy, in order to understand mechanisms of falls for potential fall prevention programmes.

## Introduction

Cerebral palsy (CP) is a complex neuro-musculo-skeletal disorder that affects movement, balance and coordination [1,2]. Children with CP experience regular falls in their day-to-day environments, with 35% of children reporting daily falls in a previous study [3]. These falls regularly occur throughout childhood, up to 18 years old, and into adulthood [3,4], unlike typically developing children who develop the autonomous ability to maintain balance and avoid falls past the age of 6–7 years old [5]. Falls throughout childhood for children with CP can have negative effects on psychosocial wellbeing such as feelings of embarrassment and reduced activity participation [6]. Understanding real-world causes and experiences of falls, from the perspectives of children with CP is vital. However, the lived experience of how children with CP experience falls in their environments is not currently reported. Typical walking analyses for children with CP are carried out over level ground and therefore overlooks uneven environmental challenges that may increase real-world falls or fall risk [7].

Children can be considered as experts of their own experiences [8]. One method that can generate rich insights into children’s lived experiences is the walk-along interview method [9]. Walk along interviews involve taking a walk while talking about day-to-day experiences, by answering questions that are prompted by environmental surroundings [10]. This method allows the recall of past experiences in familiar places, and for children, is suggested to create a more informal environment by reducing the power imbalance both between researcher (adult) and participant (child) [10]. This means a hierarchical relationship is less likely to develop which avoids children attempting to give the ‘right’ answer rather than their preferred answer [10,11]. A key reason for using walk along interviews is to gain rich information about people in their environment, both through interviewing and observing [10]. This method is particularly insightful for children since it offers children the opportunity to share experiences through their body language and gestures in response to the surrounding environment [9].

Previous walk-along interviews have explored neighbourhood walking environments and pedestrian practices with older adults [12–14]. One study used walking interviews with adults with post-injury walking disabilities [15], which revealed deep understanding of how each person experienced their disability day-to-day, including examples of how walking is performed in certain areas. Furthermore, walk-along interviews have been undertaken with children both inside the home environment [16] and outside, in the playground [17] or in the local neighbourhood [9,18]. These interviews offer insights into the lives and experiences of children and how they interact and view environments around them. Despite this, to our current knowledge this interview method is yet to be used with ambulatory children with CP to explore real-world experiences of falls and the challenges that children with CP face in their typical real-world environments.

In patient and public involvement and engagement (PPIE) work, we sought the opinions of ambulatory children with CP and parents of ambulatory children with CP, to co-develop a walk-along interview method specifically tailored for children with CP. We conducted PPIE with eight children and six parents, who helped co-design the study presented in this paper. Following the collaborative consultation with children and parents, a name was developed for the study: ‘The Walk-Along Project’ [19].

The aim of The Walk-Along Project was to explore the real-world (natural and/or built) environments that ambulatory children with CP find challenging and determine those that might cause a fall or increase fall risk based on their lived experiences.

## Methods

### Participants

Participants were recruited through various charitable organisations or schools in Northwest England to take part in a walk-along interview. Children were eligible to participate if they were between 7 and 16 years old, had a diagnosis of spastic cerebral palsy (Gross Motor Function Classification System (GMFCS) level I – II), were able to walk without walking aids, able to understand English and have adequate vision and good hearing capabilities, as judged by gatekeepers and/or parents or guardians. Children were excluded if they had any other orthopaedic or neurological condition that may alter their ability to walk. GMFCS was self-reported by parents or guardians. The study was approved by University Ethical Review Committee (ref: 22/SPS/022). Three charity gatekeepers assisted with recruitment for The Walk-Along Project between 13^th^ May 2022 and 3^rd^ December 2022. All accompanying adults who took part in this study were parents, despite inclusion of either parents or guardians. Parents and children were provided with tailored information sheets before providing written informed consent and assent, respectively. The Consolidated criteria for Reporting Qualitative Research (COREQ) [20] was used to guide reporting of this study (S1 File).

### Procedure

Definitions of both a ‘trip’ and a ‘fall’ were provided to parents and children prior to starting the walk-along interview. A trip was described to children and parents as any disturbance to walking (such as tripping over an object) or loss of balance (such as a stumble), that would not lead to a fall whereby they would come to rest on the floor. This aligned with a recent definition of a ‘near fall’ in literature [21] and was informed by language used during previous PPIE [22]. A fall was defined as any stumble event causing a disturbance to balance, that results in coming to rest on the ground or floor. This was in accordance with the World Health Organization’s definition of a fall [23] and was explained in child-friendly terms.

**Fig 1:**
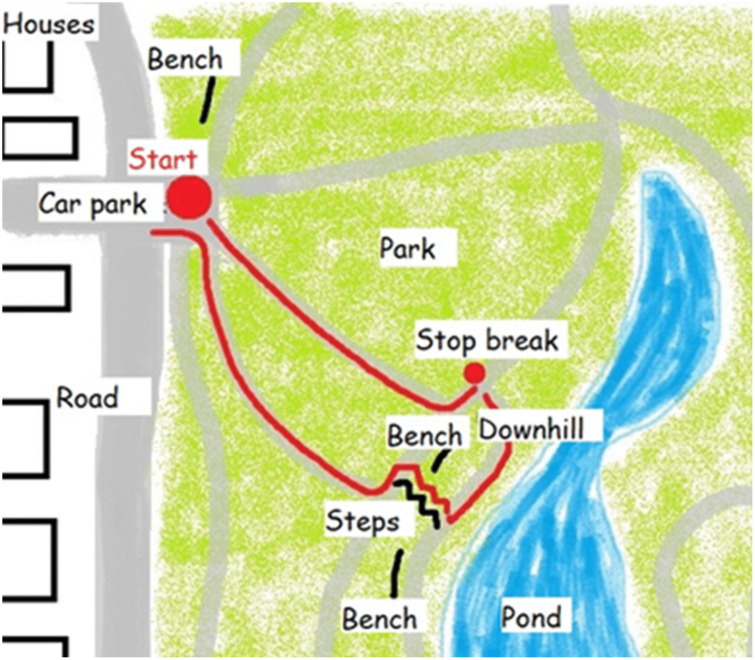
Example mud map drawn by the investigator of a walking route agreed by parents prior to a walk-along interview.

Children with CP and their parents were provided a ‘mud map’ (simplified map drawn by investigator, Fig 1) and a Google map, respectively, of a walking route predetermined by the lead investigator (RW) in a location agreed by parents during recruitment. Multiple locations were discussed during recruitment with each parent to identify a convenient, accessible place, local to their living area, for them to attend a walk-along interview. In every case, the predetermined route was chosen to represent environments that children encounter regularly in their day-to-day lives, as informed by previous PPIE and in recruitment conversations. In most cases (11 out of 12 interviews) the walking route had areas in which they had walked before on numerous occasions. Each chosen route included environments (e.g., potholes or uneven paths) that were identified during PPIE conversations that increase the risk of a trip or a fall and would therefore offer insight into children’s day-to-day lived experiences of falls. Children and parents were given the opportunity to change the walking route both prior to and/or during the walk, should anything concern them.

The walk-along interview method used in this project offered the opportunity for children, parents and investigator to engage with the surrounding environment through conversations, gestures and photo elicitation [9,24], in order to gain a well-rounded picture of environments that might cause increased fall risk to children with CP. To achieve this, The Walk-Along Project recorded conversations with microphones, captured walking environments with video cameras and captured lived experiences through child-led photo elicitation with an additional camera.

Prior to the start of the walk, the investigator used chest harnesses to attach Kaiser Baas X450 action cameras (Kaiser Baas Pty Ltd, Melbourne, Australia) to themself and to the child, if the child was comfortable. These chest mounted cameras captured videos of the environments encountered during the entire walk-along interview. Recording began by manually pressing record on each device, with default camera recording settings (capture rate: 30 fps, resolution: 4K). A third Kaiser Baas X450 camera was used in addition to video cameras for taking photos during the walk (resolution: 14MP, Field of View: 160^◦^). Two wireless RØDE GO II -clip-on microphones (RØDE Microphones, Sydney, Australia) were attached to the clothing of parents and children for high-quality audio-recording of conversations between the child, parent and lead investigator, that video cameras could not capture. Microphones were operated by the lead investigator’s Android mobile phone app, RØDE Central (Version 2.0.5, RØDE Microphones, Sydney, Australia), using default microphone settings (sample rate: 48 kHz, maximum sound pressure: 100dB).

Children and their parents walked with the investigator (RW), following the predetermined route that reflected a place they would typically walk. Children were given the map before the walk to look at the route; none of the children chose to carry the map during the walk. The investigator directed the walk and asked questions using a predetermined semi-structured interview schedule and discussion guide (S2 File). Questions included: ‘how do you feel about the walk we are taking today?’ and ‘can you see anything on this walk that might cause a trip or a fall?’. Any questions asked during the walk-along interview were first directed to children, and then parents were given the opportunity to expand on the child’s answer. Children were told they could stop the walk at any time to take photos with the third action camera of anything in the environment that they believed could cause a trip or a fall. Photos taken by children prompted further discussion in the walk-along interview. Children were given a certificate and sticker for taking part.

Following the walk-along interview, children and parents were invited to complete a two-week video diary and asked to include any videos, images, and/or written entries about places where the child might or did experience trips and/or falls in their day-to-day lives.

### Data processing

Two audio recordings (parent and child) were downloaded with RØDE Central software (Version 2.0.5, RØDE Microphones, Sydney, Australia), and then synchronised together in Adobe Audition (Version 23.3, Adobe, CA, USA). Recordings also identified any spoken conversation from the lead investigator. Two video recordings (from cameras worn by the child and investigator) were synchronised using Kinovea (Version 0.9.5, Kinovea, Charmant, J and contributors) and then matched to the synchronised audio recordings, using both a visual (hand wave on screen) and audio (‘let’s go’) cue from the investigator during the start of the walk-along interview.

Audio footage was reviewed in Microsoft Media Player (Version 11.2309.6.0, Microsoft Microsoft Corporation Washington, USA) and video footage reviewed in Kinovea, from a synchronised starting point. The lead investigator then manually transcribed interview and video diary conversations verbatim in Microsoft Word (Microsoft 365, Microsoft Corporation Washington, USA) whilst including photos and videos where appropriate of the surrounding environment. For example, if a child spoke about a particular surface, an image of that surface taken from matched video footage, was included in the transcription. Any photos captured by the children using the handheld action camera were also added to the transcription at the point of conversation in which they were taken. All photographs were taken for use in The Walk-Along Project using equipment owned and provided by the research team.

### Data analysis

Transcriptions were imported into NVivo (Version, 1.7.1, NVivo for Windows Enterprise, Lumivero, 2017) for coding by the lead investigator; two separate approaches to analysis were used.

Analytical approach 1 drew on Interpretive Description [25] and inductive coding, thus aligning with our constructivist (relativist, subjectivist, and interpretive) [26] approach, thus accepting that multiple truths exist in relation to people’s experiences and to their individual meaning-making. No *a priori* codes were used. After an iterative process we created two top level codes: ‘challenging environment’ or ‘non-challenging environment’. The code ‘challenging environment’ encompassed any environment where a child said a fall could happen. The code ‘non-challenging environment’ represented any environment where it was implied that the child felt safe or where a trip or fall would not happen. Further iterative analysis resulted in the creation of sub-codes that unpacked and provided insight into different factors related to challenging and non-challenging environments. These sub-codes helped challenge and refine the descriptors linked to the top level codes. The sub-codes allowed the analytical lens to focus on details about the types of challenging environment. For example, if a child or parent identified an uneven pavement to be a fall risk, this was coded as both a ‘challenging environment’ and ‘uneven surface’. Further sub-codes included sensory challenges, e.g., noise, vision or distractions in the environment. Additional codes were identified from commonalities across the datasets, including fall occurrences’, ‘feelings about walking’, ‘cautious behaviour’, ‘tiredness before and after walk’, ‘parent intervention’, ‘awareness of surroundings’, ‘fall mechanism’ and ‘familiarity of walking route’. As analysis deepened, codes were drawn into two themes and five subthemes (see findings).

Analytical approach 2 (frequency counting of codes) was undertaken following completion of approach 1 and was used to identify the most common challenging environments reported by the children and/or discussed during walk-along interviews. These frequency counts were considered both cumulatively and in terms of distribution across individual participants.

Children were assigned pseudonyms alphabetically in order of participation, e.g., participant 1 = Albert (Table 1).

**Table 1:** Demographics of child and summary of challenging and non-challenging environments reported during walk-along interviews.

Participant (pseudonyms)	Demographics (Sex, age, GMFCS score, parent)	Child-reported: challenging places	Parent-reported: challenging places	Child & parent-reported: non-challenging places
Albert	Boy, aged 9 years with diplegia (GMFCS I/II), Mother	- Downhill - Uneven surface (change in surface, cobbles, tree roots)	- Obstacles (e.g., branches, tree roots) - Tiredness - Concentration	- Other people - Uphill - Stepping up/down (stairs)
Ben	Boy, aged 9 years with diplegia (GMFCS I/II), Mother	- Obstacles - Uneven surface (grass, crack in path, tree roots, unseen gutter/potholes) - Slippery surface (gravel)	- Uneven surfaces - Running - Not looking - Lack of concentration	- Pavement - Flat surface - Other people
Connor	Boy, aged 11 years with diplegia (GMFCS II), Mother	- Grass - Uneven surfaces (pavement, potholes)	- Not looking and uneven surface - Stepping up/down (kerbs) - Distractions	- Downhill - Flat
Dominic	Boy, aged 16 years with right hemiplegia (GMFCS II), Mother	- Uneven surfaces (grass, raised surface, potholes) - Stepping up/down (kerbs) - Slippery surface (gravel) - Distractions	- Uneven surfaces (pavement, potholes) - Slippery surface (gravel) - Distractions	- Familiar places
Elliot	Boy, aged 14 years with diplegia (GMFCS II), Father	- Uneven surfaces (pavement and grass, grids, pebbles, potholes) - Running/fast walking - Not looking	- Stepping up (kerbs) - Uneven pavements - Noise - Non-dominant limb	- Being ‘careful’ - Stepping up/down (low kerbs, stairs) - Uphill
Freya	Girl, aged 13 years with right hemiplegia (GMFCS I) Father	- Uneven surfaces (tree roots, tactile paving, grid, stones) - Lack of awareness - Tiredness	- Non-dominant limb - Slippery surface (stones) - Tiredness	- Familiar places - Being ‘careful’ - Stepping up (tall kerbs) - Indoors
George	Boy, aged 12 years with right hemiplegia (GMFCS I/II) Father	- Uneven surfaces (potholes) - Slippery surface (gravel) and downhill - Footwear (less contact between foot and sole, e.g., wellies) - Obstacles (tree roots)	- Footwear (less contact between foot and sole, e.g., wellies) - Uneven surfaces (pavement)	- Stepping up (kerbs, stairs) - Slippery surface (mud) - Obstacles - Uneven surfaces (grass, grids, cobbles, sand) - Tiredness
Henry	Boy, aged 15 years with diplegia (GMFCS I/II), Mother	- Uneven surface (raised pavements, potholes, cobbles) - Stepping up/down (kerbs) - “When young”	- Uneven surfaces (raised pavements, potholes) - Not looking - Distractions - Noises and crowds	- If ‘careful’ - If concentrating - If taking time
Isaac	Boy, aged 15 years with right hemiplegia (GMFCS I/II), Mother	- Uneven surfaces (pavements, cobbles, potholes)	- Stepping up/down (kerbs) - Uneven surfaces (potholes, pavement/stones) - Obstacles - Vision - Tiredness - Downhill - Running	- Flat
Jasmine	Girl, aged 8 years with diplegia (GMFCS I-II), Father	- Uneven surfaces (potholes, grids) - Stepping up/down (tall kerbs)	- Foot placement and balance - Not looking - Distractions - Small obstacles - Footwear - Tiredness - Other people - Stepping up/down (kerbs)	- Walking slower - Crossing roads (concentrating) - Flat pavement - Low kerbs - Uphill
Kenny	Boy, aged 12 years with left hemiplegia (GMFCS I/II), Mother	- Unseen uneven surfaces (potholes, grass) - Stepping up/down (unseen kerbs) - Obstacles (e.g., bottle) - Downhill, uneven and running - Tiredness	- Uneven surfaces (pavement, grass, potholes) - Not looking - Distractions - Slippery surface (gravel) - Tiredness	- Flat grass
Leo	Boy, aged 10 years with right hemiplegia (GMFCS I/II), Mother	- Downhill - Uneven surface (grids, potholes, grass) - Stepping up/down (kerbs)	- Uneven surfaces (tree roots) - Stairs (no handrail) - Tiredness - Balance	- Slightly uneven grass - Smaller gravel stones

### Findings

#### Overview of key information.

Twelve ambulatory children (2 girls and 10 boys, aged between 8 and 16 years old) with CP and their parents (8 mothers, 4 fathers) took part in the walk-along interviews (Table 1). The children’s GMFCS scores ranged from I to II; six children had diplegia, and six had hemiplegia. Each walk-along interview lasted approximately 25 minutes. Two children (Dominic and Freya) returned video diaries following the walk-along interview. Dominic’s diary was a video of an uneven concrete path, with gravel and potholes, that he said would typically create a trip and fall on his walk to school. Freya spoke to the camera for three minutes about her trip or fall experiences that week.

### Presentation of themes and subthemes

**Fig 2:**
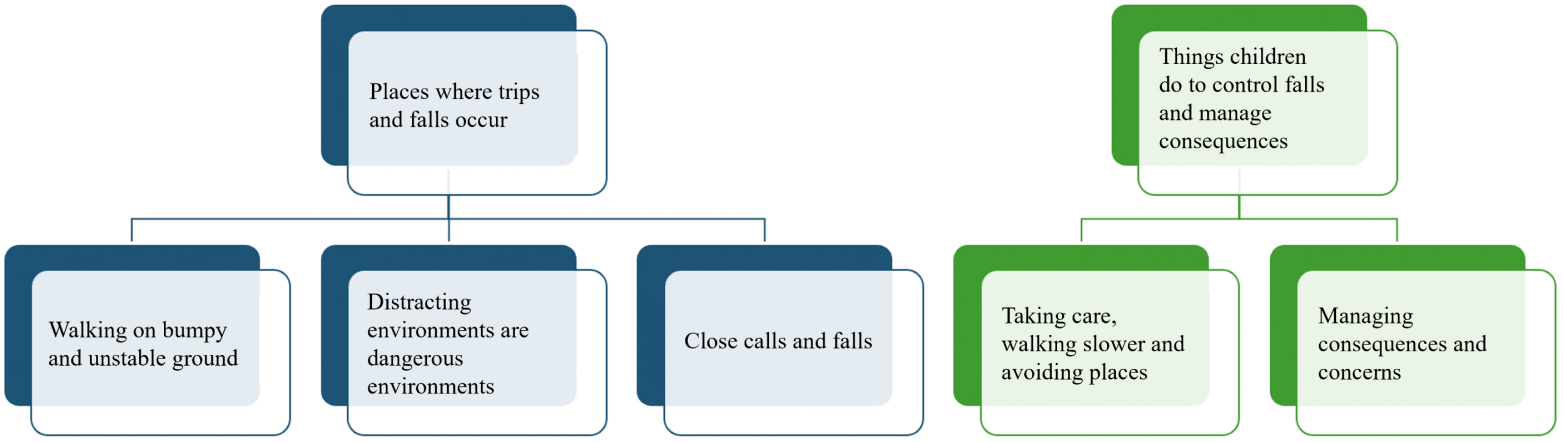
Overview of themes and subthemes.

Drawing on commonalities in data from the walk-along interviews and video diaries, two themes (‘places where trips and falls occur’ and ‘things children do to control falls and manage consequences’). were identified. These themes explored what, how and why real-world environments are challenging and cause falls or increased fall risk for ambulatory children with CP. Five subthemes were identified, three sit within the theme ‘places where trips and falls occur’ and two sit within ‘things children do to control falls and manage consequences’ (Fig 2).

### Places where trips and falls occur

#### Walking on bumpy and unstable ground.

**Fig 3:**
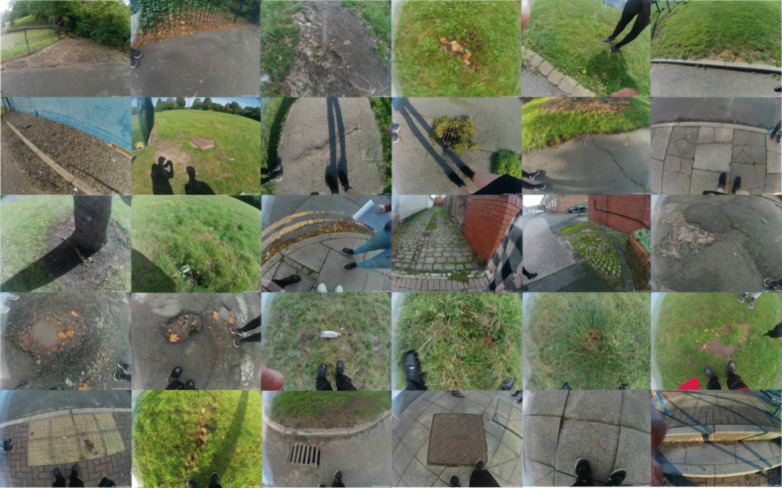
Photographs taken by participants of places they suggested could cause a trip or a fall during walk along interviews for use in The Walk-Along Project. All photographs can be seen in S1 Fig.

Photos taken by children during walk-along interviews indicate that uneven pavements and grass surfaces are the most common challenging environments that might increase falls and the risk of a trip or fall in the real-world. For example cracks in pavements or paths, tree roots under the surface of the pavement, tactile paving, cobbles, hidden potholes in the grass, ‘bumpy’ (non-level) grass, or long grass (Fig 3). These types of environments were noticed and photographed most by children on walk-along interviews, with children describing that these places are likely to cause a disturbance to balance if stepped on or they could cause a trip or stumble if unnoticed.

**Fig 4:**
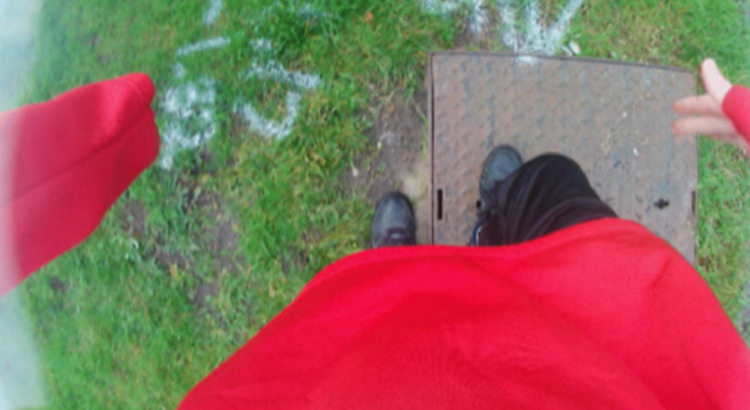
Environment talked about by George.

The most discussed challenge was uneven surfaces with all the children talking about these and typically often referring to these surfaces as “bumpy and unstable ground” (Jasmine). Freya explained why tactile paving was problematic for her “it’s all like bumpy and there’s like different levels and my foot might like get caught in one of them”. Dominic, talking of uneven ground and unexpected hazards, identified grass as a potential challenge saying “…this grass you could probably just not expect it and trip over”, and George, talking about the raised surface of a manhole cover (see Fig 4), explained:

*“Erm, it’s very uneven…so I’m just standing, I put one foot there [off the raised surface], (see photo) and one foot there [on the raised surface] it’s like boosting my other foot up, it’s very uneven” (George).*

#### Distracting environments are dangerous environments.

All the children talked about how preventative measures (taking care, walking slower and avoiding places) were much harder to implement if a distraction was present (e.g., dogs barking, other people playing football, cars driving past). Distractions caused children to ‘not look’ or ‘not concentrate’ where they were going whilst walking over an uneven surface. When walking on tactile paving, Kenny explained “if I’m not concentrating then I could stumble”. Children often described that these distractions could be auditory and/or visual. Elliot explained that he could “probably fall and trip” if “someone is shouting at me or something...and I’m talking to them and I’m not aware of something there” and Dominic demonstrated his trailing foot tripping on a raised grid by saying:

*“If I am walking in this direction and am looking at [people playing nearby] football I could go like that [catch the foot on a raised grid]…”* (Dominic).

Being able to directly see a challenging environment was described as important as if a challenging environment was obscured, e.g., a pothole in long grass, then a fall was more likely. George explained that he could “potentially fall into…potholes”, and went on to explain that this was because it may be unnoticed while walking:

*“It kind of catches you by surprise, cause you’re walking one minute, then the next your foot is just stuck in the ground”* (George).

Many previous fall experiences talked about by children with CP additionally included some form of distraction, a lack of concentration or not looking at or seeing a challenging environment. One child confirmed he had tripped on a kerb “cause I wasn’t looking” (Connor). Kenny explained that he had fallen when he was younger in a “fox hole or a rabbit hole... I accidentally put my foot on it and it felt like I kind of twisted my ankle”. He attributed this to not noticing it as he “didn’t realise it were there” and his mother added “distractions”. Another source of distraction were friends as Freya explained:

*“I was also talking to my friends so that caused me to stumble over some like roots that were coming out the ground and also some weeds that were in the cracks of the pavement, they made me trip over and like stumble a little bit”* (Freya).

#### Close calls and falls.

Eleven out of twelve children spoke at least once during walk-along interviews about their previous experiences of falls. The most common environment identified to have caused previous falls was grass potholes. Other examples included tree roots under pavement paths, uneven pavestones, obstacles, concrete potholes, gravel and cobbles.

**Fig 5:**
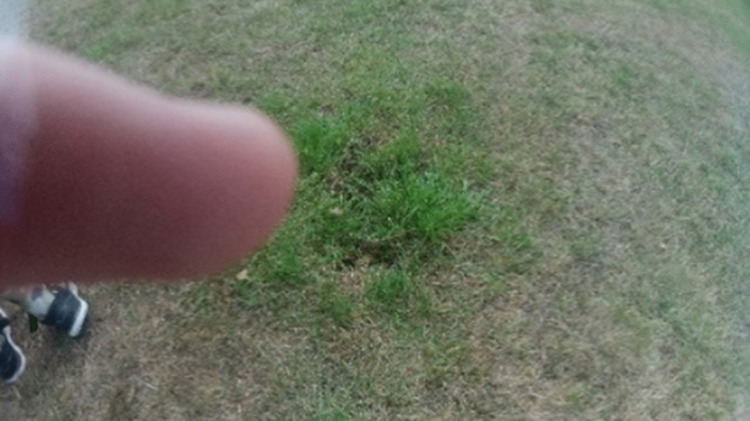
Environment talked about by Connor.

Some walks, local to where the children lived, revealed the exact places where they had fallen in the past. Connor pointed out a ditch and when asked by the investigator if someone could fall down that Connor said “I fell down that” (Fig 5) and his mother agreed “yeah you have done before. I am pretty sure we’ve been here before” (Connor’s mother).

**Fig 6:**
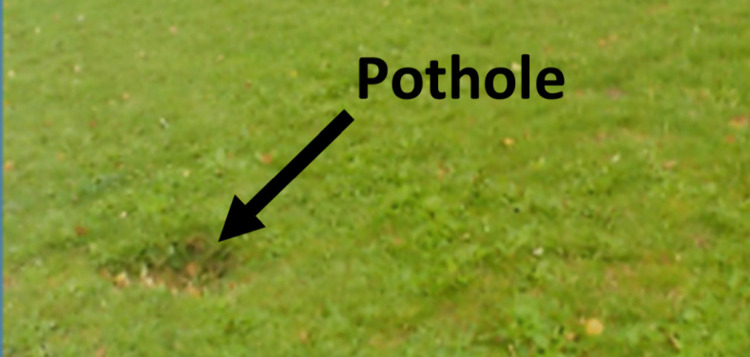
Environment talked about by Ben.

During the walk-along interviews six children experienced a trip or stumble, leading to a loss of balance that was then recovered, without leading to a fall. Two children (Connor and Jasmine) lost their balance after stepping on the edge of uneven potholes on a concrete path while trying to take a photograph in the environment (potentially the act of taking the photograph had been a distractor). Kenny became unbalanced and had to take a step backwards after stepping up onto the edge of a kerb, Leo stumbled while trying to step on an uneven grid in the grass, Henry showed instability walking over cobbles. Ben was distracted, did not see a grass pothole (see Fig 6) that he stepped into losing his balance although he recovered quickly. His mother described this as a “close call” and when asked if he had seen the hole, he replied “Nope, no clue”:

*Mother: You nearly tripped didn’t you… that was a close call*

*Interviewer (RW): What happened there?*

*Ben: I don’t know*

*Mother: You were looking at the boy on the scooter*

*Ben: No, I was looking at the camera*

*Interviewer (RW): Ahh, you didn’t see the hole? (see photo)*

*Ben: Nope, no clue.*

### Things children do to control falls and manage consequences

#### Taking care, walking slower and avoiding places.

Walk-along interviews revealed that children undertake three preventative behaviours to reduce the risk of a fall in challenging environments and these all involved children becoming aware of a potential challenge and also recognising that these actions might not prevent a fall. The actions were ‘taking care’, ‘walking slower’ and ‘avoiding places’. Taking ‘care’ about walking on an uneven grass surface for Elliot meant “obviously, I have to take my time with it a bit…and obviously be careful, but I might trip”. Several children talked of slowing down, “I’d just go slow on a grass surface and hope I don’t fall” (Jasmine). Avoidance was a strategy the children talked about, with a typical statement being “If I see an uneven bit, I’ll try to swerve [avoid] it” (Dominic).

For younger children, taking care was also more apparent through parent intervention to prevent their children falling, compared to older children. This intervention included parents suggesting behavioural ways they would intervene when walking outside: “I’ll grab his hand” (mother of Albert) and included comments made during the walk-along interview such as “be careful” (mother of Connor). Dominic’s mother explained how specifically they would intervene when Dominic was younger:

*“When they’re younger they’re not aware of what their surroundings are so all the time I [used to] say…watch this”* (Dominic’s mother).

Although more common in younger children, parent intervention was also apparent from some parents of older children. Isaac’s (aged 15) mother told Isaac to “watch where you’re going” during the walk-along interview, and Elliot’s (aged 14) father warned “watch that can there” when Elliot nearly walked into an obstacle (drink can on floor).

Older children were able to take more care in their challenging environments through greater awareness of surroundings (e.g., knowing where to slow down or be careful). This was more common in older children who were able to look back and see how their situational awareness had improved, for example “when I was younger, I didn’t use to [look at the floor], and I’ve just figured out that I need to look at the floor to know where I’m going” (Henry). However, better situational awareness of hazards did not necessarily mean that trips could be avoided, Elliot explained:

*“Obviously when you’re younger you don’t know what’s a hazard so you might run into it but now, I might, well I’m still not the best with it, but I might be able to, like I can recognise what I’m more likely to trip over”* (Elliot).

**Fig 7:**
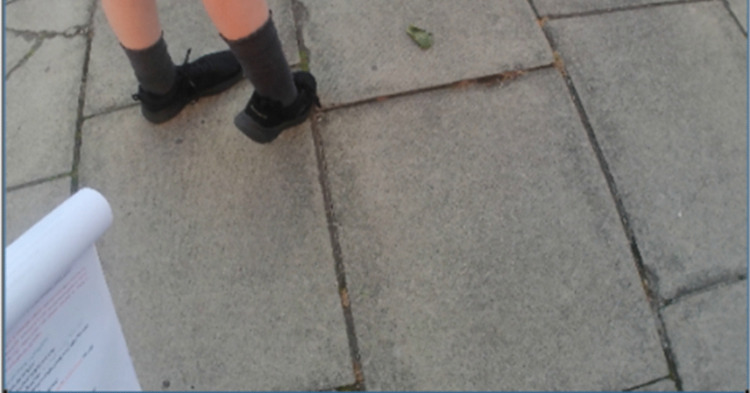
Environment talked about by Freya.

Older children showed additional awareness of how a fall might happen in challenging environments. Scenarios described by older children included tripping by catching their trail limb on a raised uneven surface, a disturbance to balance due to poor foot placement on an uneven surface and slipping on gravel. Freya demonstrated (Fig 7) how she might trip “if I’m like scraping my foot across then I would probably go like that and trip over”.

#### Managing consequences and concerns.

Children seemed comfortable talking about their regularly experienced real-world falls often coming across as being quite relaxed about them and seeing them as just part of everyday life. There were no conversations that implied specific attitudes toward falling. However, some children and/or parents discussed the impact that falls can have on day-to-day life, for example, missing school or gaining an injury. One mother recalled that “a couple of times he’s set off to school and he’s come back because he’s fallen” (Dominic’s mother). A typical response from a child regarding how falls can increase pain and injury came from Jasmine who explained:

*“Cause when I was like really little I was like falling all the time like, mostly in school I was like on the playground and I didn’t have any tights on and I was like, I was always hurting my knees”* (Jasmine).

Another example described by Leo showed a reduced feeling of safety in a challenging environment, describing a scenario where in school there is “a grass area” and “rocks that we can play on”. However, he then described that he is “a little bit cautious of going round there because they could probably hurt me”. Another example in challenging environments was described later after coming across an uneven grid during the walk-along interview:

*“This [environment] could do pretty bad damage because it has these little [bumpy bits] on it…it could probably hurt you seriously, so, let’s not try and do that”* (Leo).

This reduced feeling of safety was further evidenced by Dominic, a boy with hemiplegia, who spoke repeatedly about how their right (affected) arm, would bend at the elbow, as a physical response to heightened anxiety in challenging environments:

*“Every single time it triggers, like my brain triggers ‘oh wait an uneven surface’, my right arm goes up straight away”* (Dominic).

### Frequency of codes

**Fig 8:**
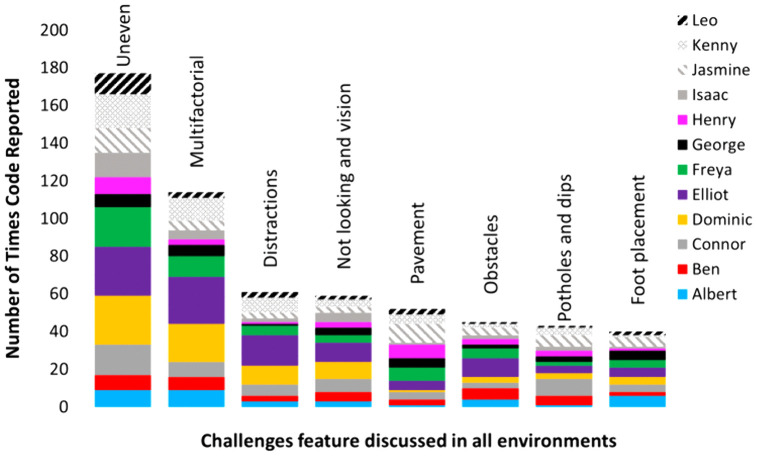
The frequency of top codes relating to challenging environments reported across all 12 interviews. Legend shows distribution of code for each participant.

Uneven environments were discussed the most across all walk-along interviews. The distribution of uneven surface codes for each participant is shown in Fig 8. The second most common code across all interviews was ‘multifactorial’, which indicated when more than one challenging environment could contribute to falls and increased fall risk. For example, an uneven surface with a distraction present. All children reported challenging environments that involved ‘pavement’, ‘obstacles’, ‘potholes or dips’ and reported that ‘distractions’, ‘not looking and vision’ or ‘foot placement’ might increase their fall risk in a challenging environment. In 11 interviews, ‘grass’, ‘downhill’, ‘kerbs’, ‘balance’, ‘stepping up or down’, ‘vision and uneven surfaces’ and ‘distractions and uneven surfaces’ were discussed with reference to challenging environments that might cause falls and increase fall risk in the real-world.

## Discussion

This is the first study to capture rich, robust qualitative data based on the lived experiences of ambulatory children with CP using a walk-along interview technique about the types of real-world challenging environments that might increase falls or fall risk. This detailed insight extends previous work regarding falls in children with CP [3]. Previous work has shown the high frequency of falls experienced day-to-day, but lack the detail into specific environments that cause additional perturbations in walking that increase falls in ambulatory children with CP. This work further adds novelty in its approach to investigating falls in ambulatory children with CP, compared to typical human movement approaches.

### Places where trips and falls occur

The Walk-Along Project now provides robust child-centred evidence for the importance of considering environmental distractions within real-world challenging walking environments when trying to understand the causes of increased fall risk in ambulatory children with CP. In previous laboratory-based challenging environments, ambulatory children with CP show cautious walking behaviours to compensate for instability [27–31]. Furthermore, our systematic review found little evidence assessing links between challenging environments in laboratory settings (e.g., uneven surfaces) and fall risk in ambulatory children with CP [32]. The Walk-Along Project now provides detailed evidence, through photos and lived experiences, of the types of environments, specifically uneven surfaces with distractions, that increase fall risk for ambulatory children with CP. Potentially the link between challenging environments and fall risk was previously limited, because laboratory environments were not underpinned by lived experiences of children with CP that account for the types of uneven environments and distractions where they fall most.

Furthermore, The Walk-Along Project provides novel evidence of the importance of distractions to real-world fall risk. Work is yet to explore how stability and fall risk may be affected over a challenging environment (real-world or laboratory based) with a distraction present. Children with bilateral CP have shown minimal differences in walking parameters with an additional distraction or visual stimulus during routine gait analysis in a laboratory over level ground [33,34]. Work with older adults demonstrates that in the real-world, attention is moved away from a walking path ahead, and toward other people in the environment, whereas in a laboratory setting attention was focused to the walking path ahead [35]. The Walk-Along Project, perhaps, mirrors this finding revealing that in real-world settings ambulatory children with CP divert their attention from the walking path, although as seen in previous studies [33,34] this does not occur in the laboratory over level ground.

### Things children do to control falls and manage consequences

Children with CP walking slower and more carefully in the real-world aligns with previous laboratory work. To increase walking stability and try to prevent a fall, children with CP walk slower compared to typically developing children over level ground and over uneven surfaces in laboratory environments [36,37]. This cautious behaviour might link to an increased anxiety or concern of the consequences of falling (e.g., injury) and reduced balance confidence in these challenging environments. In a previous study, children with CP have demonstrated reduced feelings of safety when negotiating steep inclines and declines, as shown by increased focus, less talking and more gaze focus towards the floor [28] and shown reduced balance confidence compared to typically developing peers [6]. In the current study, this was demonstrated by children through physical responses (Dominic) or discussion of pain previously caused by falls (Jasmine) and concern around potential consequences of falls in challenging environments (Leo). In real-world challenging environments, this reduced feeling of safety may actually be linked to increased fall risk, because less attention is available for the task [38].

The Walk-Along Project also revealed that when children become distracted, they are less likely to implement cautious behaviours to reduce fall risk (taking care, walking slower and avoiding places), because their attention and vision is moved away from looking where they were walking. Thus, as suggested by children, if a distraction is present in a challenging environment, the number of falls and fall risk increases as reflected in high real-world fall frequency [3]. This finding supports the argument that cautious behaviours implemented by ambulatory children with CP are conscious actions rather than habitual behaviour [39,40], since distractions interrupt the action of being more cautious in challenging environments [41,42]. This was identified by both younger (e.g., Ben) and older (e.g., Elliot) children during The Walk-Along Project. Perhaps this supports the use of behavioural interventions, for example, learning to maintain cautious behaviours when in highly distracted environments (e.g., taking care at the park or when walking with other people), to create more habitual cautious behaviours when in challenging environments that increase fall risk [43].

Many fall experiences discussed during walk-along interviews were due to children not looking where they were going or due to an environmental distraction. Vision is an important factor into maintaining balance for children with CP. When walking, vision is used in a feedforward manner, by looking two steps ahead to plan a walking path [44]. Therefore, when children with CP are distracted, this causes a disruption in visual processing of the challenging environment ahead. This disruption may then cause any anticipatory adjustments to even small perturbations, as suggested in The Walk-Along Project, to not be implemented, leading to a trailing limb causing a trip, or a misplaced foot step. Although all children are likely to become distracted in some environments, this issue is particularly pertinent for children with CP. Children with CP already show balance deficits and visual impairments compared to typically developing children [45,46], therefore compensatory mechanisms that may be interrupted are more important for maintaining stability. Furthermore, the response to a trip or loss of balance may be impaired in children with CP compared to typically developing children, as demonstrated in previous studies with standing perturbations [47], therefore increasing the likelihood of a fall following a trip or stumble.

### Implications for future practice and fall prevention

The Walk-Along Project offers implications for developing protocols that replicate real-world walking environments that include audio and visual distractions, as this may improve understanding of mechanisms of falls and fall risk in ambulatory children with CP. In doing so, distractions could be incorporated into regular assessments and implemented within the community to identify ambulatory children with CP who might be at high fall risk. Such insights could inform future fall prevention programmes. Moreover, The Walk-Along Project supports views previously indicated in the literature [30,48] that visual factors that contribute to falls need further investigation. Future work or practical interventions on scanning strategies may help children identify hazards in a distracted environment.

Individual differences in children should also be considered when determining which children may be at highest fall risk and therefore benefit most from such interventions. For example, although children and parents were not asked to disclose coexisting conditions, Dominic identified that he often becomes distracted when walking and that his diagnosis of ADHD, might contribute. Isaac’s mother explained that Isaac’s vision is impaired, thus she has to provide regular parent intervention. An implication from this is the importance of identifying children who might be at high fall risk due to multiple, individualised factors. A further key takeaway from this work is the poor quality of everyday environments. Albeit specific in this study to the local region, potholes, broken pavements and similar factors are hazards to most people, especially children and those with CP.

Another important potential outcome from The Walk-Along Project lies in the development and use of wearable technology for children with CP for assessing falls and fall risk in the real outdoor world. The development of sensors and markerless technologies could enable information about fall and fall risk behaviours to be captured in the real-world, which could inform future fall prevention programmes.

### Strengths and limitations

The first strength of this work is that the methods have been informed by careful and detailed PPIE, with children with CP and their parents [19]. Prior PPIE informed an applicable and feasible walk-along interview technique, tailored for ambulatory children with CP, which was used in this study. This early work ultimately makes The Walk-Along Project grounded in the thoughts and opinions of children with CP from conception to implementation. The second strength of this work is the pioneering use of the walk-along interview technique with ambulatory children with CP for investigating real-world falls. This extends the population of children who have been involved in interviews and specifically, walk-along interviews, as previous studies have been confined to typically developing children [9,16–18,49]. We show that this method is acceptable and safe for ambulatory children with CP and provide practical recommendations.

This study is limited by a relatively small sample size. Despite reaching data saturation and previous work showing similar samples with children [9,16], a larger number of participants may have generated stronger themes related to age and experience of falls. Furthermore, child participants were all ambulatory without the use of walking aids. Ambulatory children with CP are suggested to experience the most falls [3], however lived experiences captured by The Walk-Along Project might not generalise to children with less functionality and those requiring walking aids. Another limitation is the low response of participation in video diaries (n = 2) to supplement the walk-along interviews. Greater participation in the video diary component of the study may have provided more detailed insight into specific examples of falls and challenging environments. Finally, thoughts and feelings relating to falls, which informed the subtheme ‘managing consequences and concerns’, were not the primary focus of these walk-along interviews. Thus, few conversations occurred during walk-along interviews about how children felt when they fell and those that did occur, were not in-depth.

## Conclusion

This is the first study to explore the lived experiences of ambulatory children with CP in challenging environments using walk-along interviews to investigate real-world fall risk. The Walk-Along Project revealed novel insights about environments that are challenging, cause falls or increased fall risk and the preventative behaviours that children use to avoid falls in their real-world environments. The influence of a distracting environment was an important factor linked to high fall occurrence and fall risk in the real-world. This is vital information for understanding mechanisms of falls in day-to-day environments, informing future fall prevention programmes such as training protocols over “bumpy and unstable ground” and thus targeting the negative psychosocial factors associated with increased falls. Finally, it seems fitting in this child-centred study that we turn to a child to provide a concluding statement. Leo (age 10yrs), upon beginning the walk-along interview and being asked ‘*how do you feel about the walk today’*, compassionately shared:

*“[This walk], will help you and people who don’t have CP understand how people who have CP…fall*”.

(Leo)

## Supporting information

S1 File Consolidated criteria for Reporting Qualitative Research (COREQ). (PDF)

S1 Fig All photographs taken by children with CP during walk-along interviews. (Note: All photographs used with full consent/assent. All equipment owned and provided by the research team).
(PDF)

S2 File Interview schedule and discussion guide. (PDF)
