# Supplementary material for: “I’d probably trip over it because it’s bumpy”: A qualitative exploration of the lived experiences of ambulatory children with cerebral palsy walking in challenging environments
Source: PLoS One. 2025 Dec 3;20(12):e0337316. doi: 10.1371/journal.pone.0337316 (PMC12674536; doi:10.1371/journal.pone.0337316)

S1 Fig. *All photographs taken by children with CP during walk-along interviews. (Note: All photographs used with full consent/assent. All equipment owned and provided by the research team).*

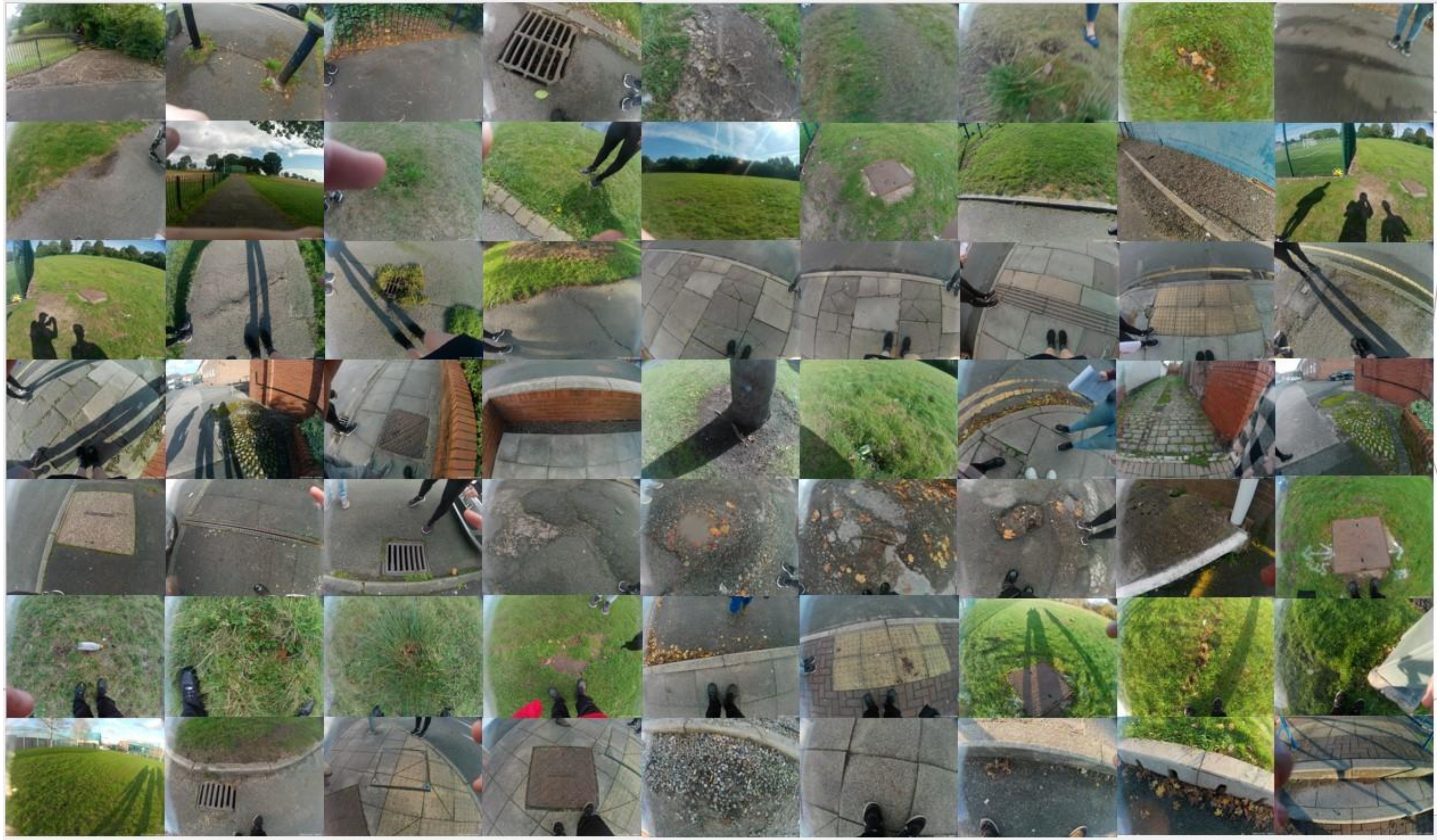

Supplement: S1 Fig — (Note: All photographs used with full consent/assent. All equipment owned and provided by the research team). (PDF) [file pone.0337316.s002.pdf]
