# Supplementary material for: “I’d probably trip over it because it’s bumpy”: A qualitative exploration of the lived experiences of ambulatory children with cerebral palsy walking in challenging environments
Source: PLoS One. 2025 Dec 3;20(12):e0337316. doi: 10.1371/journal.pone.0337316 (PMC12674536; doi:10.1371/journal.pone.0337316)
Supplement: S2 File — (PDF) [file pone.0337316.s003.pdf]

S3 File. *Interview schedule and discussion guide*

Interview Schedule

All the statements and questions during the interview will be tailored in a language appropriate for the age of each child participant (ages 7-16 years) and parent/guardian participants.

Introduction and gaining consent

- Interviewer and child and parent/guardian participants meet in pre-arranged location.
- Saying hello and re-introducing (if necessary).
- Summary of study, using Participant Information Sheets (given to participants again) as a guide and opportunity for children and parents/guardians to ask questions, specific focus on:
  - Brief overview of the walk and walk-along interview process (and data handling)
  - Explain we want both children's and parent's/guardian's views but we will firstly direct questions to children for parents/guardians to follow up after.
  - Google map and mud map of route given to parent/guardian and child.
  - Pre-walk statement (directed to child but involving parent/guardian):

"This is the walk we are planning to do (refer to map provided and any known landmarks). The reason we want to take a walk today is to talk about the places where you find it easy to walk and the places that might make you worried about tripping or falling. You are the expert so anything you tell me will help me learn more about how falls happen and how that makes you feel.

I may ask us to stop occasionally and talk about where we are on our walk. You can stop any time too.

Your (parent/guardian) will be with us the whole time. We can take rests during the walk whenever you want, if you feel tired just let me or (parent/guardian) know. We can also make the walk shorter if that's helpful.

  - Show child and parent/guardian camera and recording equipment

"If you consent, we would also like to record the walks with cameras and microphones. I will be wearing one (show camera) and if you (child) would like to wear one too that would be great. If not, we will ask (parent/guardian) to wear one. The microphones will be attached with a clip (show microphones), and you will have a couple of minutes to get used to the equipment before we get going. The recording will continue until otherwise said after the walk."

  - Introduction of video diaries and video diary sharing platform

"We would also like to capture home video diaries about trips or falls that you may encounter day-to-day that we may not come across during our walk today. This is completely optional and additional to the walk-along interview. It would require you taking pictures or videos during your day-to-day life and uploading them to a secure University OneDrive folder"

  - Offer option of multiple walks if necessary - ask parent/guardian
  - No right or wrong answers, no need to participate, just want to learn from their thoughts
  - Confidentiality, anonymity, withdrawal from study and withdrawal of data

"Any information on cameras, paper and recorders will be confidential and only used for research. Whatever you say along the walk will be anonymised, which means no one will know it is you, even if names are mentioned during the walk."

- Any questions?

Informed consent and assent obtained from parent/guardian and child

Collection of child demographics (Age, GMFCS and/or CP diagnosis)

Set up recording equipment

- Cameras on interviewer using chest strap
- Children given the option to wear chest strap video
- If child doesn't want to, parent/guardian will be asked to wear the camera
- Children given a third camera during the interview to take pictures at stop points
- Microphones set up and attached to children and parents/guardians where possible
- Provide 2-minute familiarisation for cameras and microphones
    - "Are you OK to wear the camera and start the walk?"

Start recording

Start walk (with stops and checks as necessary)

Adjust map where necessary (and note down any reasons for changes)

Follow interview discussion guide

Stop Recording

Post-walk reminders

- Offer the option of another walk if necessary
- Reminder of video diaries and how to take part and upload to OneDrive (direct to instructions on participant information sheet previously given out)

Final thanks and summary

- Thank child and parent/guardian for their involvement
- Child offered certificate and/or sticker

Interview Discussion Guide

Language will be tailored for the age of each participant and guided by environmental cues and responses, using this guide as a reference. All questions may not necessarily be asked from this discussion guide or in the order as specified. Questions will firstly direct towards children with opportunity throughout the interview for parents/guardians to follow up on answers given by children.

How do you feel about where we are walking today?

1. Have you taken walks like this before?
   a. Do you feel okay about taking this walk today?
   b. How much energy do you have?
   c. Do you feel like you may lose your balance or fall on this walk/in this place?
2. Have you ever fallen here before?
   a. What might make you fall/not fall here?
      i. Prompts: functional, environmental, sensory, intrinsic (next page)

Have you ever tripped or fallen in a place like this?

1. If **NO**:
   a. Do you often fall or lose your balance balance/feel wobbly when you are walking?
   b. What places where you usually walk might cause a fall?
      i. How is that different to where we are walking now?
2. If **YES**:
   a. Did you trip or fall?
   b. Where did you fall/trip?
   c. What made you lose your balance and trip/fall over?
      i. Prompts: functional, environmental, sensory, intrinsic (next page)
   d. Would you ever not go somewhere because you may fall over? – Why?
      i. What makes you decide to not go somewhere?
      ii. What would you normally do if you wanted to avoid somewhere?
      iii. Would this change if you were with different people?
   e. How do you feel if you fall over?
   f. Do you think about falling a lot when you are walking outside?
   g. How often do you trip and not fall over?
   h. If talking about a trip, restart question: Have you ever **fallen**?

Are there any things that you can see and hear now that might make you fall?

i. Cars, playing, dogs, football etc.

Are there things that you do that make you fall or lose balance more often?

ii. What activities?
i. Why? - Prompts: functional, environmental, sensory, intrinsic (next page)

Stop Point

Take a look around, is there anything that makes you feel at risk of a fall?

2. If **NO**:
    a. Keep referring back to this
3. If **YES**:
    a. "Take a picture of what you can see around us that could make you fall"
    b. Discuss these pictures
    c. Why would this make you fall?
        i. Prompts: functional, environmental, sensory, intrinsic (see below)
    d. Would you experience this day-to-day?
        i. Has anything like this [whatever they identify] made you fall before?
        ii. Tell me about these

Prompts for why trips or falls occur:

1. Why?
2. Prompts:
    a. *Environmental* (Others, surface, obstacles, foot placement, step up/down, inclines, weather)
    b. *Functional* (Holding on, running, picking up feet, footwear/AFO's, fatigue)
    c. *Sensory* (Spatial awareness, noises, vision, vestibular)
    d. *Intrinsic* (Distractions, confidence, concentration)
3. Why would/wouldn't any of these make you fall?

Final reflective questions

(asked at the end of the walk outside with appropriate resting place e.g. bench)

How did you feel about the walk we took today?

1. Was there anywhere you thought was particularly tricky to walk?
2. Was there anywhere you felt you were more likely to trip or fall?
3. Do you feel okay now that we have taken the walk?
4. How much energy would you say you have now?
5. Do you feel like you are likely to fall now?
6. Has this changed since the start of the walk?

Is there anything else that you think would cause a fall that we haven't talked about?

Anything else you want to say about falls or the walk?
